# Effect of coculturing canine notochordal, nucleus pulposus and mesenchymal stromal cells for intervertebral disc regeneration

**DOI:** 10.1186/s13075-015-0569-6

**Published:** 2015-03-14

**Authors:** Irene TM Arkesteijn, Lucas A Smolders, Sandra Spillekom, Frank M Riemers, Esther Potier, Björn P Meij, Keita Ito, Marianna A Tryfonidou

**Affiliations:** Department of Biomedical Engineering, Eindhoven University of Technology, P.O. Box 513, 5600 MB Eindhoven, The Netherlands; Department of Clinical Sciences of Companion Animals, Faculty of Veterinary Medicine, Utrecht University, PO Box 80.154, NL-3508 TD Utrecht, The Netherlands; Clinic for Small Animal Surgery, Vetsuisse Faculty, Zurich University, Winterthurerstrasse 260, CH-8057 Zurich, Switzerland; Laboratoire de Bioingénierie et Biomécanique Ostéo-Articulaire (B2OA), UMR CNRS 7052, Université Denis Diderot Paris 7, Sorbonne Paris Cité, 690 Paris, France; Department of Orthopedics, University Medical Center Utrecht, P.O. Box 85500, HP G05.228 3508 GA, Utrecht, The Netherlands

## Abstract

**Introduction:**

Early degenerative changes in the nucleus pulposus (NP) are observed after the disappearance of notochordal cells (NCs). Thus, it has been suggested that NCs play an important role in maintaining the NP and may have a regenerative potential on other cells of the NP. As the number of resident NP cells (NPCs) decreases in a degenerating disc, mesenchymal stromal (stem) cells (MSCs) may be used for cell supplementation. In this study, using cells of one species, the regenerative potential of canine NCs was assessed in long-term three-dimensional coculture with canine NPCs or MSCs.

**Methods:**

Canine NCs and canine NPCs or MSCs were cocultured in alginate beads for 28 days under hypoxic and high-osmolarity conditions. Cell viability, cell morphology and DNA content, extracellular matrix production and expression of genes related to NC markers (*Brachyury*, *KRT18)* and NP matrix production (*ACAN*, *COL2A1*, *COL1A1*) were assessed after 1, 15 and 28 days of culture.

**Results:**

NCs did not completely maintain their phenotype (morphology, matrix production, gene expression) during 28 days of culture. In cocultures of NPCs and NCs, both extracellular matrix content and anabolic gene expression remained unchanged compared with monoculture groups, whereas cocultures of MSCs and NCs showed increased glycosaminoglycan/DNA. However, the deposition of these proteoglycans was observed near the NCs and not the MSCs. Brachyury expression in the MSC and NC coculture group increased in time. The latter two findings indicate a trophic effect of MSCs on NCs rather than vice versa.

**Conclusions:**

No regenerative potential of canine NCs on canine NPCs or MSCs was observed in this study. However, significant changes in NC phenotype in long-term culture may have resulted in a suboptimal regenerative potential of these NCs. In this respect, NC-conditioned medium may be better than coculture for future studies of the regenerative potential of NCs.

**Electronic supplementary material:**

The online version of this article (doi:10.1186/s13075-015-0569-6) contains supplementary material, which is available to authorized users.

## Introduction

Low back pain is the most common cause of disability worldwide; in many cases, it is attributable to intervertebral disc (IVD) degeneration [1]. Current therapies for low back pain are symptom-oriented and are successful in relieving pain. However, they do not preserve the function of the IVD, and, in the long term, the results of treatment are suboptimal. Because the first signs of disc degeneration are observed in the core of the disc, called the nucleus pulposus (NP), regeneration of the NP is of great interest for designing new therapies to maintain the function of the IVD [2].

Degeneration of the IVD involves the transition from a gelatinous to a fibrotic NP [3]. This change is associated with a decreased ability of the NP to convert compressive forces into evenly distributed tensile stresses in the surrounding annulus fibrosus (AF), with consequent degeneration of the AF [4]. Both the resident cells and extracellular matrix of the NP undergo major changes in this degenerative process. At birth, a human NP is populated by clusters of large, vacuolated notochordal cells (NCs) and by small, chondrocyte-like nucleus pulposus cells (NPCs), whereas a degenerating NP is populated by increasingly apoptotic NPCs and possibly fibrochondrocyte-like cells [5]. Furthermore, the healthy gelatinous NP is rich in proteoglycans that keep the tissue hydrated, whereas in the degenerated NP, matrix contains less proteoglycans, different collagen types and more matrix-degrading enzymes [5].

Interestingly, degenerative changes are observed after loss of NCs. Similarly to humans, chondrodystrophic dog breeds show loss of NCs early in life. These dogs develop generalized IVD degeneration as young adults and are predisposed to develop subsequent IVD diseases, such as herniation, later in life [6-8]. Conversely, non-chondrodystrophic dog breeds maintain gelatinous NPs, rich in NCs, far into adulthood and generally develop IVD diseases only at an advanced age and in isolated locations in the spine [9]. Therefore, it has been suggested that NCs play an important role in maintaining the NP by synthesizing new matrix [10] and by regulating matrix synthesis of other cells [11]. Because of the fact that NPC + NC cocultures and cultures of NPCs in NC-conditioned medium have resulted in a significant increase in proteoglycan production by NPCs [11-13], NCs are interesting targets for research strategies to regenerate the NP.

As the number of NPCs decreases in a degenerating disc, it may also be necessary to complement the resident cell population to maintain the health of the NP matrix. Repopulation of the degenerating disc with healthy NPCs would be a logical strategy. However, harvesting NPCs is likely to induce degeneration of donor discs. Alternatively, autologous mesenchymal stromal (stem) cells (MSCs) are readily available, show high proliferation rates and can differentiate into NPC-like cells [14,15]. MSC cultures in NC-conditioned medium have resulted in increased proteoglycan synthesis [16], and, when injected into NPs, MSCs showed increased matrix production and proliferation [17,18].

It is known that NCs, MSCs and NPCs cocultured in different combinations have a certain regenerative potential [11,13]; however, owing to varying culture conditions, previous studies have not allowed a valid comparison of the regenerative potential of NCs cocultured with NPCs with NCs cocultured with MSCs. Therefore, in this article, we present the first long-term study of coculture of canine NCs with NPCs or MSCs, within the same species. Articular chondrocytes, with a phenotype similar to but ECM production different from NPCs [19], were simultaneously cocultured with NPCs or MSCs to assess NC-specific effects in coculture. We hypothesized that canine NCs stimulate the production of extracellular matrix by canine NPCs and MSCs when cultured in three dimensions and under near-physiological NP conditions (approximately native cell density, hypoxia and adjusted osmolarity). To evaluate the regenerative effect of NCs on NPCs and MSCs in coculture, we compared the effect of NCs on (1) MSCs, with articular chondrocytes (ACs) as a control group; (2) NPCs, with ACs as a control group; and (3) NPCs, with MSCs as a control group.

## Methods

### Cell isolation and expansion

All cells used in this study were obtained from healthy dogs killed for unrelated experiments, which were approved by the ethics committee on animal experimentation of Utrecht University (DEC: 2012.III.05.046). Experiments were conducted with freshly isolated NCs and cryopreserved, bone marrow-derived MSCs, NPCs and ACs.

Complete spines (cervical, thoracic and lumbosacral regions) were collected from four mongrel dogs (non-chondrodystrophic (NCD1 through NCD4); female, age 1.3 ± 0.5 years, weight 26.0 ± 14.6 kg (mean ± standard deviation (SD)). The technique of harvesting NPs has been developed by experienced, board-certified veterinary surgeons (BM and MAT) who are familiar with IVD-related spine surgery in canine patients with IVD diseases. The technique was optimized to expose and retrieve 26 discs (6 cervical, 13 thoracolumbar and 7 lumbar IVDs) within the time span of 1 hour in a canine cadaveric spine. Care was taken to include only NP tissue and not AF or endplate tissue. NP tissue was pooled per donor in Dulbecco’s modified Eagle’s medium (DMEM)/Ham’s F-12 (GlutaMAX; Life Technologies, Carlsbad, CA, USA) + 2% penicillin/streptomycin (P/S; PAA Laboratories, Cölbe, Germany). The pooled NPs were digested according to the method used by Smolders *et al*. [20]: 0.1% pronase (Roche Diagnostics, Almere, The Netherlands) for 45 minutes and 0.05% collagenase type II (Worthington Biochemical, Lakewood, NJ, USA) overnight, both at 37°C. Subsequently, the cell suspension was filtered with a 40-μm cell strainer (BD Biosciences, Erembodegem, Belgium), and cells >40 μm (mostly clusters) were flushed away from the strainer surface with culture medium and collected. After centrifugation (at 500 *g* for 5 minutes at room temperature), cell cluster pellets were resuspended in 100% fetal bovine serum (FBS) (FBS Gold; PAA Laboratories). Per dog, 26.0 ± 12.3 × 10^6^ (mean ± SD) living cells were counted in a propidium iodide (PI) assay (Nucleocounter NC-100; Chemometec, Nieuwegein, The Netherlands). Fluorescent PI can bind double-stranded DNA but is unable to permeate the membrane of living cells. In this assay, the number of viable cells was determined by calculating the difference between the number of dead cells in suspension before (dead cell concentration) and after lysis of the cell membranes (total cell concentration, including clustered cells).

MSCs, NPCs and ACs were harvested from eight Beagle dogs (chondrodystrophic (CD1 through CD8; male, age 2.0 ± 0.3 years, weight 12.0 ± 1.3 kg (mean ± SD)). For each donor, bone marrow was collected and MSCs were isolated as described elsewhere [21]. When 80% confluence was reached (within 7 days), MSCs were cryopreserved at P0.

Cervical and thoracic spines were collected, and NPs were harvested and pooled per donor as described above for NC isolation. ACs were obtained from both stifle joints. After the joint was opened, cartilage was harvested from the distal femoral condyles, the patella and the proximal tibial plateau. NPs and knee cartilage were digested in 0.15% pronase for 45 minutes and 0.15% collagenase type II overnight, both at 37°C. The cell suspension was filtered with a 70-μm cell strainer (BD Biosciences), and the NPCs and ACs were collected from the filtrate by centrifugation. The yield per dog was 7.0 ± 3.0 × 10^6^ living NPCs and 14.2 ± 3.6 × 10^6^ living ACs (mean ± SD). The cells were cryopreserved directly after isolation (P0). MSCs, NPCs and ACs were thawed and expanded 6 days before the isolation of NCs. MSCs were cultured up to passage 2, whereas NPCs and ACs were cultured up to passage 1. All three cell types were cultured in high-glucose (4.5 g/L) DMEM (Life Technologies) + 10% FBS (Greiner Bio-One, Alphen aan den Rijn, The Netherlands) + 1% P/S (Lonza, Basel, Switzerland).

### Experimental design

To compare the stimulation potential of NCs, NCs were cocultured with MSCs or NPCs separately. In order to identify whether the observed effects were NC-specific, ACs were used in place of NCs in the same combinations. Monoculture controls for each individual cell type were also conducted. Finally, the effect of MSCs on NPCs in coculture was also examined (Table 1). For each experiment repetition, multiple MSC, NPC and AC donors were pooled, and different combinations of MSCs, NPCs and ACs were used for each NC donor (Table 2). The number of repetitions for each cell group is shown in Table 1. Alginate beads of these cell combinations were made as previously described for semisolid beads by Guo *et al*. [22] using a 26-gauge needle and 1.2% w/v alginate (Sigma-Aldrich, St Louis, MO, USA). The cell concentration for monocultures was 3 × 10^6^/ml alginate and 6 × 10^6^/ml alginate for cocultures (Table 1). The cell concentration was doubled in cocultures to assess the effect of the regulatory cells. The alginate beads were cultured in agarose-coated (to avoid cell adherence) well plates at 37°C, 5% CO_2_ and 5% O_2_ (hypoxia) for 28 days. The medium consisted of high-glucose DMEM + 5% FBS + 1% sodium pyruvate (Lonza) + 1% P/S + 1% 0.4 M KCl (Merck, Darmstadt, Germany) + 1% 5 M NaCl (Merck) and was changed twice weekly. The latter two medium components were used to adjust the osmolarity of the medium to 400 mOsm/L, similar to the osmolarity in healthy bovine NP tissue [23,24].

**Table 1:** **Experimental groups, cell combinations, cell concentrations and repeats** ^**a**^

**Group**	**Cell types**	**Cell concentration (×10** ^**6**^ **/ml alginate)**	**Number of repeat experiments**
1	NC	3	3 (4 for day 1)
2	MSC	3	4
3	NPC	3	4
4	AC	3	4
5	MSC + NC	3 + 3	3 (4 for day 1)
6	NPC + NC	3 + 3	3 (4 for day 1)
7	NPC + MSC	3 + 3	2
8	MSC + AC	3 + 3	4
9	NPC + AC	3 + 3	3

^a^NC, Notochordal cell; MSC, Mesenchymal stromal cell; NPC, Nucleus pulposus cell; AC, Articular chondrocyte.

**Table 2:** **Cell pooling for each experimental repetition** ^**a**^

**Repetition**	**NC donor**	**MSC donors**	**NPC donors**	**AC donors**
1	NCD1	CD2,4,8	CD2,5	CD2,4
2	NCD2	CD2,8	CD6,7,8	CD2,4
3	NCD3	CD1,2	CD3,4	CD4,5
4	NCD4	CD3,8	CD6,7,8	CD2,4

^a^NC, Notochordal cell; MSC, Mesenchymal stromal cell; NPC, Nucleus pulposus cell; AC, Articular chondrocyte; CD, Chondrodystrophic; NCD, Nonchondrodystrophic.

### Assessments

Samples were analyzed on days 1, 15 and 28 for cell viability and morphology, proteoglycan production and gene expression.

To assess cell viability, alginate beads were incubated for 1 hour in 10 μM calcein-AM and 10 μM PI (both from Molecular Probes, Eugene, OR, USA) in phosphate-buffered saline. Cells were imaged using a confocal microscope (CLSM 510 META NLO; Carl Zeiss, Sliedrecht, The Netherlands). The beads were assessed at a depth range of 50 μm to 200 μm from the bead surface.

To assess cell morphology and matrix deposition, alginate beads were fixed in 3.7% formalin (Merck) + 100 mM CaCl_2_ (Calbiochem, Darmstadt, Germany). After ethanol dehydration, the beads were embedded in paraffin. Subsequently, 5-μm-thick paraffin sections were cut (RM2255; Leica, Rijswijk, The Netherlands) and stained with hematoxylin and eosin (cell morphology) or Safranin O/Fast Green/hematoxylin to assess matrix deposition. The sections were examined by light microscopy (BX60; Olympus, Zoeterwoude, The Netherlands) and a color charge-coupled device camera (Leica).

Three alginate beads per time point were digested overnight at 60°C in 450 μl of papain digestion buffer (100 mM phosphate buffer; 5 mM ethylenediaminetetraacetic acid, disodium dihydrate; 5 mM l-cysteine hydrochloride, anhydrous; and 125 to 140 μg/ml papain (all from Sigma-Aldrich) at pH 6.0) for biochemical analysis. The DNA content was measured with a Hoechst dye assay [25] using calf thymus DNA (Sigma-Aldrich) as a reference. With the same solution, the sulfated glycosaminoglycan (GAG) content was measured using a modified dimethylmethylene blue assay [26] (pH 1.5) using shark cartilage chondroitin sulfate (Sigma-Aldrich) as a reference. GAG values were normalized to the DNA content (GAG/DNA). The DNA was expressed per alginate bead. The hydroxyproline (HYP) content, as a measure for collagen, was determined in a chloramine-T assay [27] using *trans*-4-hydroxyproline (Sigma-Aldrich) as a reference. The HYP content was below detection limits in all samples.

Cells were isolated from five alginate beads per group by a 5-minute incubation in sodium citrate digestion buffer (55 mM sodium citrate, Sigma-Aldrich; 0.15 M NaCl, Merck; 25 mM 4-(2-hydroxyethyl)piperazine-1-ethanesulfonic acid, Sigma-Aldrich) for gene expression analysis. Subsequently, cells were lysed in 300 μl of Buffer RLT (RNeasy Mini Kit; Qiagen, Leusden, The Netherlands) + 1% β-mercaptoethanol (Sigma-Aldrich) and stored at −80°C until RNA isolation. Total RNA was isolated using the RNeasy Micro Kit (Qiagen) according to the manufacturer’s instructions. The quantity and purity of RNA were determined with a spectrophotometer (NanoDrop ND-1000; Thermo Fisher Scientific, Wilmington, DE, USA). Subsequently, the iScript cDNA Synthesis Kit (Bio-Rad Laboratories, Veenendaal, The Netherlands) was used to reverse-transcribe the total RNA. Gene expression was analyzed in a quantitative RT-PCR (qPCR) (CFX384, Bio-Rad Laboratories) experiment using iQ SYBR Green Supermix (Bio-Rad Laboratories). Relative quantification was calculated using the comparative threshold cycle (2^−ΔΔ*C*t^) algorithm with kinetic PCR efficiency correction [28], with the results normalized to the reference gene and day 1 of the same gene. Relative gene expression was measured for the reference genes ribosomal protein S19 (*RPS19*), TATA box-binding protein (*TBP*) and glyceraldehyde 3-phosphate dehydrogenase (*GAPDH*), and the target genes: (1) NC markers *brachyury* and cytokeratin 18 (*KRT18*) [19] and (2) matrix production-associated genes aggrecan (*ACAN*); collagen, type I, α1 (*COL1A1*); collagen, type II, α1 *(COL2A1*); primer sequences (Additional file 1).

### Data analysis and statistics

Statistical analyses were performed using R statistical software [29]. Linear mixed models [30], containing both fixed (culture duration and cell group) and random effects (individual dog), were used to analyze the described parameters separately for the qPCR and GAG/DNA analyses. A random intercept for each dog was added to each model to take the correlation of the observations within a dog into account. The Akaike Information Criterion was used for model selection. If necessary, models were optimized by correcting for unequal variances and/or for autoregressive correlation. Conditions for the use of mixed models, including normal distribution of the data, were assessed by analyzing the residuals (probability–probability and quantile–quantile plots) of the acquired models. No violations of these conditions were observed.

In order to compare increases in DNA content between culture groups and time points, differences in DNA content between different time points in culture were calculated for all groups and expressed as percentage changes. These values were used as readout parameters in the above-described models.

Because cell culture groups were composed of different cell types and cell combinations, GAG production was normalized by cell number (calculated as GAG/DNA ratio). Using this parameter, the GAG production between different time points was compared within all culture groups. Also, GAG/DNA values were normalized to values obtained at day 1 in order to compare different cell culture groups in their increase or decrease in time. These values were used as readout parameters in the statistical models as described above.

For the gene expression analysis, we first evaluated whether the expression of genes of interest differed significantly between different groups on day 1 in culture. For this purpose, the Δ*C*_t_ value for individual target genes at day 1 in culture was used. To evaluate the effect of combing two cell types on the gene expression of relevant genes, the change for each culture group relative to its own expression on day 1 (as different culture groups showed significant differences in baseline gene expression on day 1) was statistically assessed using the parameter ΔΔ*C*_t_. ΔΔ*C*_t_ was calculated for both days 15 and 28 in culture and was defined as (Δ*C*_t_ (day 15) − Δ*C*_t_ (day 1)) and (Δ*C*_t_ (day 28) − Δ*C*_t_ (day 1)) for days 15 and 28 in culture, respectively.

With the aim of answering the research questions, *P*-values were calculated for the above-described parameters for the following comparisons:

- To assess the effect of NCs on MSCs, with ACs as control group: Groups compared were NC, MSC, AC, MSC + NC, and MSC + AC.
- To assess the effect of NCs on NPCs, with ACs as control group: Groups compared were NC, NPC, AC, NPC + NC, and NPC + AC.
- To assess the effect of NCs on NPCs, MSCs as control group: Groups compared were NC, MSC, NPC, MSC + NC, NPC + NC, and MSC + NPC.

For all the described models, the Benjamini-Hochberg method [31] was used to correct for multiple comparisons. *P* < 0.05 was considered statistically significant.

## Results

### Notochordal cells in monoculture

**Figure 1:**
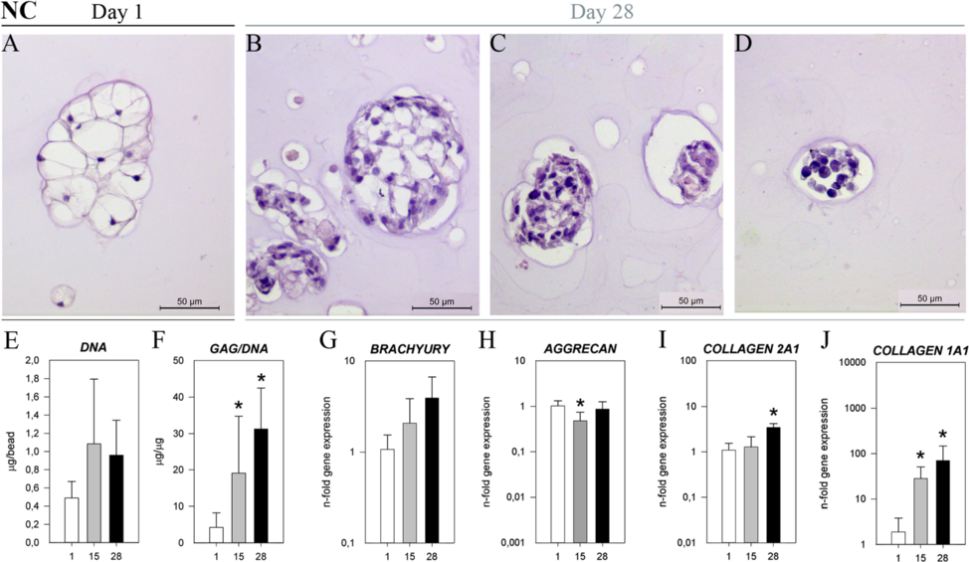
**Notochordal cells in culture.** Histopathological slides (hematoxylin and eosin (H&E)) of **(A)** notochordal cells (NCs) on day 1, H&E of NCs on day 28 with different morphologies; **(B)** NC-like clusters with vacuoles; **(C)** fibroblast-like clusters; and **(D)** small, round cells in clusters (scale bar = 50 μm for H&E). **(E)** DNA content, **(F)** glycosaminoglycan content normalized to DNA (GAG/DNA), and gene expression of **(G)** brachyury; **(H)** aggrecan; **(I)** collagen, type II, α1 (collagen 2A1); and **(J)** collagen, type I, α1 (collage 1A1). White bars = day 1, gray bars = day 15, black bars = day 28. *P < 0.05, significantly different from day 1.

On day 1, large, vacuolated NCs were primarily organized in clusters (Figure 1A). The size of the vacuoles and the size of NC clusters decreased with time, especially during the first 2 weeks. At day 28, three NC morphological phenotypes could be observed: (1) in clusters, with smaller vacuoles than on day 1; (2) in clusters of fibroblast-like cells; and (3) occasionally in small clusters of round cells (Figure 1B,C and D, respectively).

**Figure 2:**
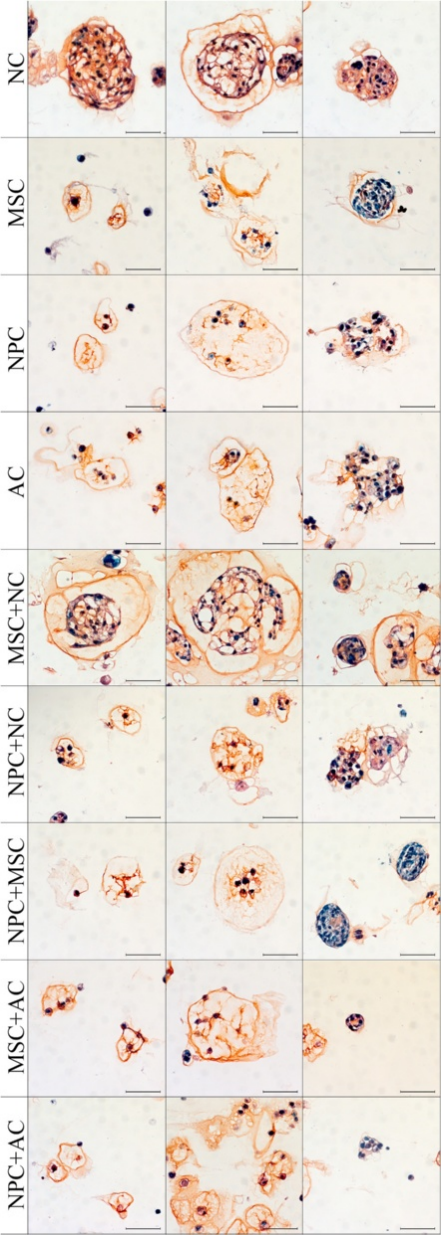
**Extracellular matrix deposition.** Histopathological slides of typical cell morphologies on day 28 of notochordal cells (NCs), mesenchymal stromal cells (MSCs), nucleus pulposus cells (NPCs), articular chondrocytes (ACs), MSC + NC, NPC + NC, NPC + MSC, MSC + AC, and NPC + AC. Prior to staining, alginate was removed with sodium citrate. Cell nuclei are stained blue (hematoxylin), proteoglycans are red (Safranin O) and collagen is green (Fast Green) (scale bar = 50 μm).

On day 28, more GAG was found surrounding the vacuolated NCs than the fibroblast-like NCs (NC row in Figure 2). NC viability was high on day 1 (Additional file 2). GAG/DNA increased significantly with time (Figure 1F, Additional file 3), consistent with Safranin O/Fast Green staining. The gene expression of *ACAN* decreased significantly on day 15, but thereafter it returned to values found at day 1 of culture. The expression of both *COL2A1* and *COL1A1* increased significantly over time (Figure 1H,I,J, respectively, and Additional file 4). Furthermore, the expression of NC markers *brachyury* and *KRT18* remained stable over 28 days (Figure 1G, Additional files 4 and 5).

### The regulatory effect of notochordal cells on mesenchymal stromal (stem) cells in coculture

**Figure 3:**
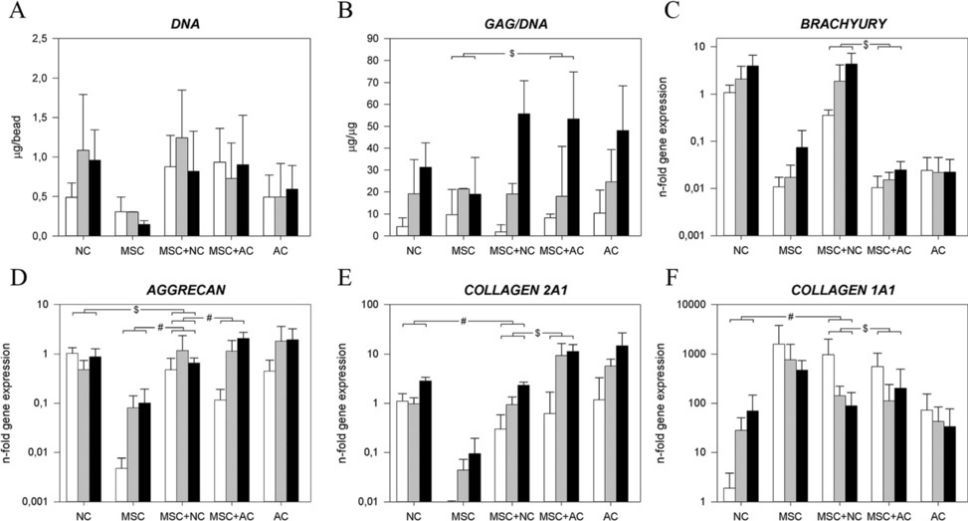
**Notochordal cells and mesenchymal stromal cells in coculture (control: articular chondrocytes).** Depiction of the **(A)** DNA content and **(B)** glycosaminoglycan (GAG) content normalized to DNA (GAG/DNA) and the relative gene expression of **(C)** notochordal cell (NC) marker brachyury; **(D)** aggrecan; **(E)** collagen, type II, α1 (collagen 2A1); and **(F)** collagen, type I, α1 (collagen 1A1). White bar = day 1, gray bar = day 15, black bar = day 28. ^$^ *P* < 0.05, significant difference in the increase in GAG/DNA production/gene expression in time between groups. ^#^ *P* < 0.001, significant difference in the increase in gene expression in time between groups. Only relevant comparisons are displayed. MSC, Mesenchymal stromal (stem) cell; AC, Articular chondrocyte.

On day 28, morphologies of cocultured NCs, MSCs and ACs were the same as each individual cell type in monoculture (Additional file 6). The cell viability was high on day 1 (Additional file 2) and the DNA content within all culture groups remained statistically unchanged over time (Figure 3, Additional file 3).

In each group, the amount of GAG/DNA increased significantly with time, with the MSC + AC group showing a significantly higher increase than the MSC group (Additional file 3). This was in line with the Safranin O staining, which showed that MSCs + ACs produced more proteoglycans than MSCs alone (Figure 2). Although not statistically significant, the average GAG/DNA in the MSC + NC group on day 28 was notably higher compared with the MSCs and NCs monoculture groups. This effect was also seen in the Safranin O staining, which showed that the NCs produced more proteoglycans in the presence of MSCs (Figure 2).

The *brachyury* gene expression was significantly higher in the NC and MSC + NC groups than in the other groups. *KRT18* expression remained stable in all culture groups (Additional file 5). The *ACAN* and *COL2A1* expression of NCs increased least of all groups over time, and the expression of *ACAN* and *COL2A1* in the MSC + AC group increased significantly more with time than the MSC + NC group (Figure 3D,E). Although *COL1A1* expression in the NC group increased with time, coculture of MSC + NC showed a significantly greater decrease than the MSC + AC group (Figure 3F). Additional files 7 and 8 how in detail the statistical differences in gene expression levels between groups on day 0 and during culture.

### The regulatory effect of notochordal cells on nucleus pulposus cells in coculture

**Figure 4:**
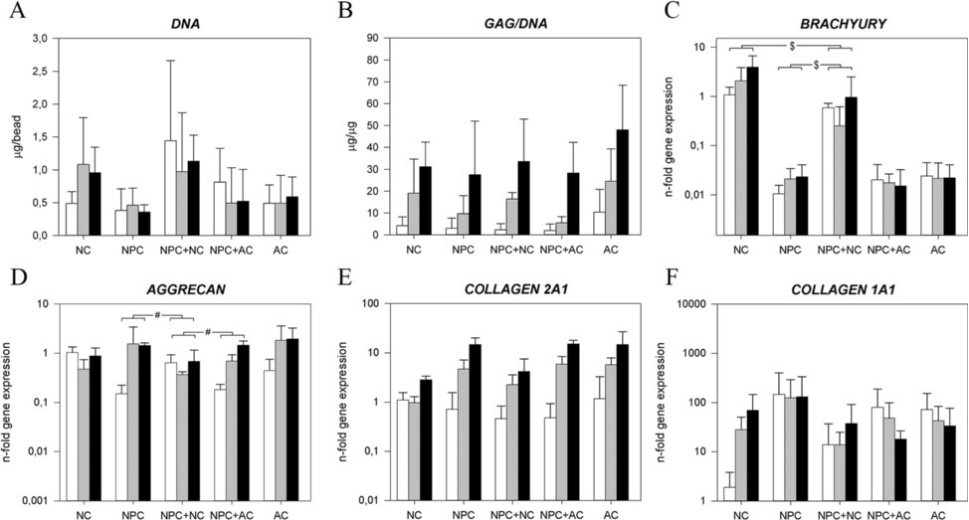
**Notochordal cells (NCs) and nucleus pulposus cells (NPCs) in coculture (control: articular chondrocytes (ACs)).** Depiction of the **(A)** DNA content and **(B)** glycosaminoglycan (GAG) content normalized to DNA (GAG/DNA) and the relative gene expression of **(C)** NC marker brachyury; **(D)** aggrecan; **(E)** collagen, type II, α1 (collagen 2A1); and **(F)** collagen, type I, α1 (collagen 1A1). White bar = day 1, gray bar = day 15, black bar = day 28. ^$^ *P* < 0.05, significant difference in the increase in gene expression in time between groups. ^#^ *P* < 0.001, significant difference in the increase in gene expression in time between groups. Only relevant comparisons are displayed.

The morphology of the NPCs on day 1 and 28 was similar to that of the ACs (Additional file 6). On day 28, morphologies of the cocultured cells were the same as in monocultures. The DNA content of all culture groups remained unchanged over time (Figure 4A), and the amount of GAG/DNA increased significantly and similarly in all groups (Figure 4B, Additional file 3). Safranin O/Fast Green staining indicated that the cells in cocultures of NPC + NC and NPC + AC deposited amounts of proteoglycans similar to the monocultures of the respective cell types (Figure 2).

*Brachyury* gene expression was significantly higher in the NC and NPC + NC groups at day 1 compared with the other groups, but expression increased significantly more in NCs alone than in coculture with NPCs (Figure 4C). *KRT18* expression remained stable in all culture groups (Additional file 5). On day 1 in culture, NCs showed a significantly higher *ACAN* gene expression than NPCs. NPC + AC showed a significantly higher increase in *ACAN* expression in comparison to NPC + NC. When we compared NPC, NC, NPC + NC, and NPC + AC groups, we observed no significant differences in changes in *COL2A1* and *COL1A1* expression (Figure 4E and F). Additional files 7 and 8 show in detail the statistical differences on gene expression level between groups on day 0 and during culture.

### The regulatory effect of notochordal cells on nucleus pulposus cell vs. notochordal cells on mesenchymal stromal (stem) cells

**Figure 5:**
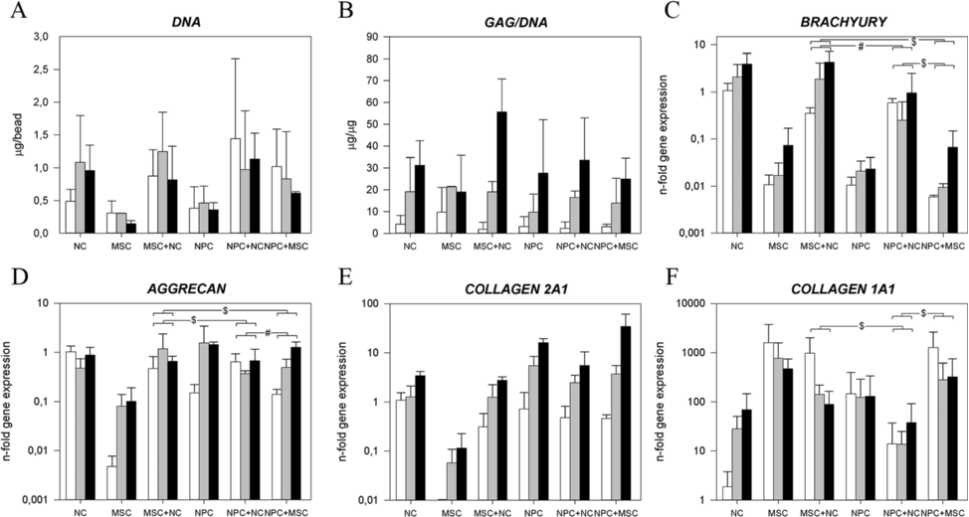
**Comparison of the effect of notochordal cells (NCs) on nucleus pulposus cells (NPCs) and mesenchymal stromal cells (MSCs).** Depiction of the **(A)** DNA content and **(B)** glycosaminoglycan (GAG) content normalized to DNA (GAG/DNA) and the relative gene expression of **(C)** NC marker brachyury; **(D)** aggrecan; **(E)** collagen, type II, α1 (COL2A1); and **(F)** collagen, type I, α1 (COL1A1). White bar = day 1, gray bar = day 15, black bar = day 28. ^$^ *P* < 0.05, significant difference in the increase in gene expression in time between groups. ^#^ *P* < 0.001, significant difference in the increase in gene expression in time between groups. Only relevant comparisons are displayed.

When NPCs were combined with MSCs, the DNA content did not change significantly (Figure 5A). Unlike the high GAG/DNA of the MSC + NC group compared with monocultures, there was no synergistic effect on GAG production in the NPC + NC and NPC + MSC groups (Figure 5B, Additional file 3). These findings were confirmed by Safranin O/Fast Green staining.

*Brachyury* expression increased significantly more in the MSC + NC and MSC + NPC groups than in the NPC + NC group (Figure 5C). *ACAN* expression increased less in the NPC + NC group than in the MSC + NC group, but both increased less than in the NPC + MSC group (Figure 5D). *COL2A1* expression was not significantly different between MSC + NC and NPC + NC in time. Finally, *COL1A1* decreased more in the MSC + NC and MSC + NPC groups than for NPC + NC (Figure 5F). Additional files 7 and 8 show in detail the statistical differences in gene expression levels between groups on day 0 and during culture.

## Discussion

### The regenerative potential of notochordal cells

Cell-based regenerative strategies for the NP have used both NPCs and MSCs [32], but with limited success. NCs, involved in disc development, have been suggested to stimulate the regenerative capacity of the resident cells of the degenerated disc (NPCs), as well as the additionally introduced exogenous MSCs [33]. Previous short-term (3 days) NPC + NC coculture and culture of bovine NPCs in medium conditioned by canine NCs resulted in significantly increased proteoglycan metabolism [11]. The same was observed for human MSCs cultured in porcine NC-conditioned medium [16,34]. With an increased duration of NPC + NC coculture (14 days; bovine + porcine), still a slight, but significant increase in GAG/DNA compared with monoculture groups was observed [13]. However, in a long-term study (28 days), no effect of porcine NCs on the GAG production of a bovine NPC/MSC mix (ratio 1:1) was observed [35]. In the latter study, it was hypothesized that the change in phenotype of the NCs, as observed during the culture period, negatively affected the regenerative potential of these cells.

In the present study, an *in vitro* coculture model was used to evaluate whether NCs could enhance the regenerative potential of NPCs and MSCs. ACs were employed as a control for NC-specific effects. Despite the small number of repeats, addition of NCs to MSCs in coculture did result in substantially more GAG/DNA compared with MSCs alone, but this was probably a result of increased GAG production by the NCs rather than of the NCs stimulating the MSCs. Similarly, when ACs were added to MSCs, more GAG/DNA was produced, but, again, this was through production by the ACs. When NCs, MSCs or ACs were added to NPCs, there was no stimulatory effect of coculture and the results were similar to those with NPCs alone. Thus, in this long-term, canine three-dimensional coculture system, there was no stimulation of NPCs or MSCs cocultured with other cells.

### Loss of notochordal cell phenotype in long-term culture

Despite the fact that in the present study monocultures of NCs maintained their *brachyury* and *KRT18* expression at basal levels, the phenotype of the NCs changed during culture. Initially, all NCs contained large vacuoles and were organized in clusters, whereas at the end of the culture period three different populations of clustered NCs were observed: NCs with smaller vacuoles, fibroblast-like cells and small clusters of round cells. The total amount of cells in the NC cultures increased slightly in time, but the percentage of vacuolated NCs, which resemble the original population, decreased. Unlike the observation by Potier *et al*. that porcine NCs produced negligible amounts of proteoglycans during culture for 28 days [35], the canine cells in the NC group in the present study produced significant amounts of proteoglycans accompanied by significantly increased expression of *COL2A1* and *COL1A1* in time. This difference may be due to the chosen culture conditions or to the difference in species used. The assessment of additional markers for the NC phenotype, such as *KRT8* and *KRT19* [19,36], could have improved the insight into the phenotypical change in the present study.

NCs in clusters were (co)cultured in three-dimensions, *in vitro*, under physiological hypoxia and osmolarity, with the aim of maintaining their phenotype [24,37-40]. In previous studies, NCs were cultured in DMEM/F-12 supplemented with 8% to 15% FBS [11,39]. In the present study, the basal culture medium (high-glucose DMEM) used was chosen to support the chondrogenic potential of NPCs and MSCs [41]. Excess of glucose in this culture medium may have induced a change in NC phenotype. Adjustment of the medium composition may have been detrimental to the phenotype of the NCs, as recent reports indicate that glucose deprivation, as well as high concentrations of glucose, results in decreased proliferation and increased apoptosis of NCs [37,42,43]. NCs cultured in either advanced DMEM/F-12 culture medium or minimum essential medium Eagle, alpha modification (α-MEM), have been shown to maintain their notochordal phenotype better than NCs cultured in simple DMEM/F-12 or DMEM [24,44,45]. It has been suggested that the presence of ascorbic acid in DMEM/F-12 and α-MEM is the key factor for maintaining the NC phenotype [44].

Therefore, with the aim of preserving NC phenotype in long-term culture, it is recommended to culture NCs in clusters [40] in serum-free (unpublished results) α-MEM at an osmolarity of 400 mOsm/L [24] under hypoxic conditions [39]. In addition, a physiological pH and compression could contribute to maintenance of the NC phenotype.

### Trophic effects of mesenchymal stromal (stem) cells on notochordal cells

Whereas NCs were not able to induce a response of NPCs or MSCs in coculture, the MSCs appeared to regulate an increase in GAG production and *ACAN* expression of the NCs. Only a few, single, chondrocyte-like cells could be identified in these cocultures on day 28, so it seems that only a small percentage of the MSCs (or NCs) differentiated to a chondrogenic phenotype. To the authors’ knowledge, only one report on the effect of MSCs on NC behavior exists [46]. When MSCs were injected in murine NPs, with induced degeneration, the number of resident NCs and the differentiation of the resident NCs to NPC-like cells increased significantly compared with untreated degenerated NPs. Furthermore, half of the injected MSCs differentiated to NPC-like cells after 4 weeks of culture. Altogether, this resulted in increased proteoglycan deposition by the entire cell population [46], based on the differentiation of MSCs toward NPC-like cells and their trophic effect on the resident cell population. In previous MSC + AC cocultures, of which more reports are available, similar observations were made: MSCs have mainly trophic effects on ACs in coculture, and, surprisingly, the majority of the MSCs disappears during culture. Only a minority of the MSCs differentiate to a chondrogenic phenotype, thereby contributing to the matrix production [47-49]. In this respect, the recently described niche of NP progenitor cells [50] may interact with the resident NCs and thereby play an important, but currently unidentified, role in the degenerative cascade of the IVD.

### Study limitations

The present study had some limitations. With regard to determining the fate of each cell type during 28 days in culture, the application of cell labeling (that is, with green fluorescent protein or long-term cytoplasmic staining) would have been valuable. Cell labeling would have shed a light on the final cell ratio, possible changes in cell morphology and cells secreting proteoglycans. Second, cell densities in monoculture (3 × 10^6^ cells/ml) and coculture (6 × 10^6^ cells/ml) were chosen in the range of the *in vivo* cell concentration in the human NP (4 to 5 × 10^6^ cells/ml) [51]. However, the cell densities in the coculture groups were double those of the monoculture groups, with the aim of observing the additive effect of the supposedly regenerative NCs and control cell type ACs on MSCs and NPCs. However, this discrepancy in cell density may have affected the paracrine signaling and metabolism of the cells [52].

### Future studies

We propose that NC-conditioned medium, produced in culture conditions that support the NC phenotype, is a better alternative for evaluating the regenerative effect of NCs on NPC or MSCs, as it overcomes the drawbacks of loss of NC phenotype and bidirectional intercellular communication in culture. NC-conditioned medium has been shown to increase anabolic gene expression by NPCs [11,53] or MSCs [16,33] and to inhibit apoptosis of NCs [45]. Furthermore, challenging NPCs or MSCs in *in vitro* tissue models in the presence of NC-conditioned medium would give further insight into *in vivo* regenerative processes.

## Conclusions

Direct addition of canine NCs did not have a regenerative effect on canine MSCs or NPCs in the present study design. The lack of a regenerative response may be due to a change in phenotype of the NCs under culture conditions that support the chondrogenic potential of MSCs and NPCs. This may be avoided in future studies by using NC-conditioned medium. These results have implications for strategies using NCs and MSCs for IVD regeneration.

## Additional files

Additional file 1:
**List of primers for gene expression analysis.**
Additional file 2:
**Cell viability.** Cell viability on day 1 of notochordal cells (NCs), mesenchymal stromal cells (MSC), nucleus pulposus cells (NPCs), articular chondrocytes (ACs), MSC + NC, NPC + NC, NPC + MSC, MSC + AC and NPC + AC. Cytoplasm of living cells was stained with calcein-AM (green fluorescence), and DNA of dead cells was stained with propidium iodide (red fluorescence) (scale bar = 200 μm). Additional file 3:
***P*** **-values of changes in DNA and GAG/DNA content during culture.** NS: not significant with *P* > 0.1. Additional file 4:
***P*** **-values of changes in gene expression within monoculture groups.** NS: not significant with *P* > 0.1. Additional file 5:
**Cytokeratin 18 expression.** Depiction of the relative gene expression of cytokeratin 18 in the following comparisons. **(A)** The effect of notochordal cells (NCs) on mesenchymal stromal cells (MSCs). **(B)** The effect of NCs on nucleus pulposus cells (NPCs). **(C)** The effect of NCs on NPCs vs. MSCs. White bar = day 1, gray bar = day 15, black bar = day 28. Additional file 6:
**Mesenchymal stromal cells (MSCs), nucleus pulposus cells (NPCs) and articular chondrocytes (ACs) in culture.** Histopathological slides (H&E) of **(A)** AC on day 1 (similar to NPC and MSC on day 1). **(B)** through **(D)** Day 28 H&E staining of (B) MSCs, **(C)** NPCs and (D) ACs (scale bar = 100 μm). The **(E)** DNA content, **(F)** glycosaminoglycan content normalized to DNA (GAG/DNA) and gene expression of **(G)** *aggrecan*, **(H)** collagen type 2, α1, and **(I)** collagen type 1, α1. White bar = day 1, gray bar = day 15, black bar = day 28. **P* < 0.01, significantly different from day 1. Additional file 7:
***P*** **-values of differences in gene expression between groups on day 0.** NS: not significant with *P* > 0.1. Additional file 8:
***P*** **-values of differences in change in gene expression between groups during culture.** NS: not significant with *P* > 0.1.

## Abbreviations

AC: Articular chondrocyte
*ACAN*: Aggrecan
AF: Annulus fibrosus
α-MEM: Minimum essential medium Eagle, alpha modification
CD: Chondrodystrophic
*COL1A1*: Collagen, type I, α1
*COL2A1*: Collagen, type II, α1
DMEM: Dulbecco’s modified Eagle’s medium
FBS: Fetal bovine serum
GAG: Glycosaminoglycan
*GAPDH*: Glyceraldehyde 3-phosphate dehydrogenase
H&E: Hematoxylin and eosin
HYP: Hydroxyproline
IVD: Intervertebral disc
*KRT18*: Cytokeratin 18
MSC: Mesenchymal stromal (stem) cell
NC: Notochordal cell
NCD: Nonchondrodystrophic
NP: Nucleus pulposus
NPC: Nucleus pulposus cell
PI: Propidium iodide
P/S: Penicillin-streptomycin
qPCR: Quantitative RT-PCR
*RPS19*: Ribosomal protein S19
SD: Standard deviation
*TBP*: TATA-box-binding protein
