# Supplementary figures and images for: Effect of coculturing canine notochordal, nucleus pulposus and mesenchymal stromal cells for intervertebral disc regeneration
Source: Arthritis Res Ther. 2015 Mar 14;17(1):60. doi: 10.1186/s13075-015-0569-6 (PMC4396569; doi:10.1186/s13075-015-0569-6)

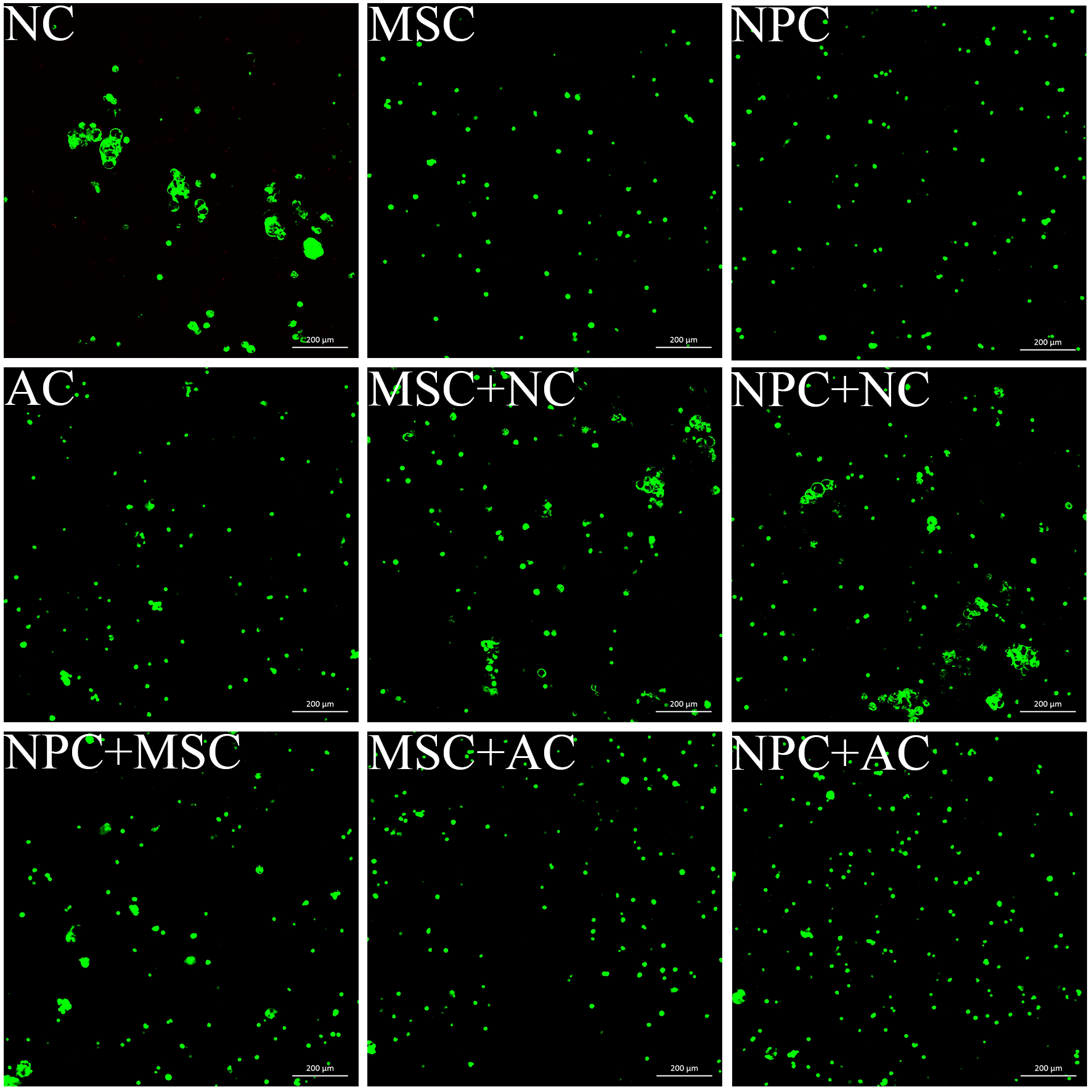

Supplement: Additional file 2: — Cell viability. Cell viability on day 1 of notochordal cells (NCs), mesenchymal stromal cells (MSC), nucleus pulposus cells (NPCs), articular chondrocytes (ACs), MSC + NC, NPC + NC, NPC + MSC, MSC + AC and NPC + AC. Cytoplasm of living cells was stained with calcein-AM (green fluorescence), and DNA of dead cells was stained with propidium iodide (red fluorescence) (scale bar = 200 μm). [file 13075_2015_569_MOESM2_ESM.tiff]

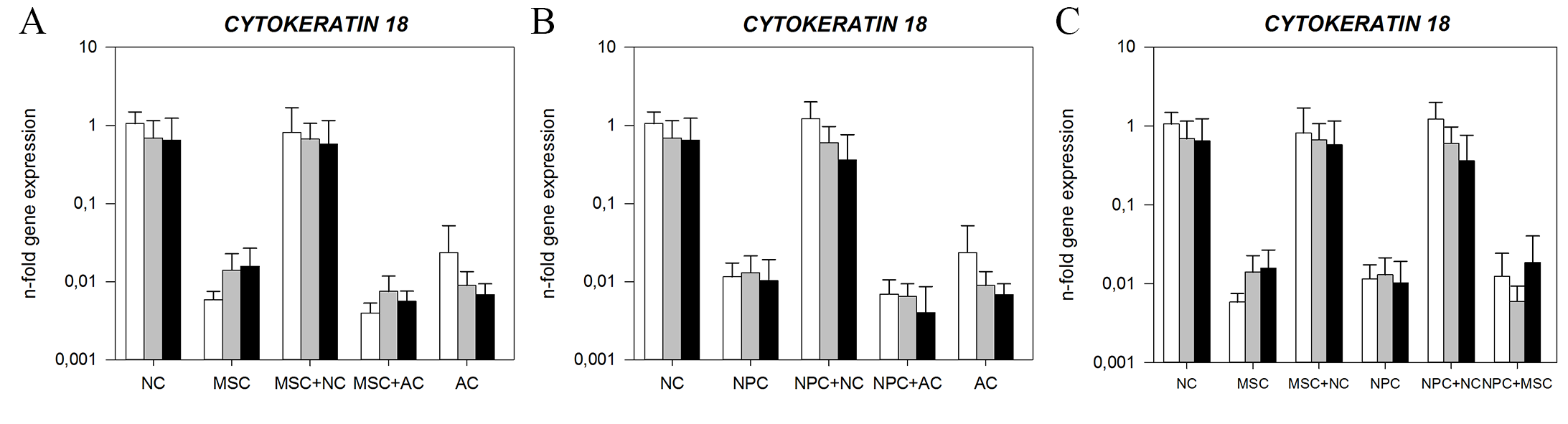

Supplement: Additional file 5: — Cytokeratin 18 expression. Depiction of the relative gene expression of cytokeratin 18 in the following comparisons. (A) The effect of notochordal cells (NCs) on mesenchymal stromal cells (MSCs). (B) The effect of NCs on nucleus pulposus cells (NPCs). (C) The effect of NCs on NPCs vs. MSCs. White bar = day 1, gray bar = day 15, black bar = day 28. [file 13075_2015_569_MOESM5_ESM.tiff]

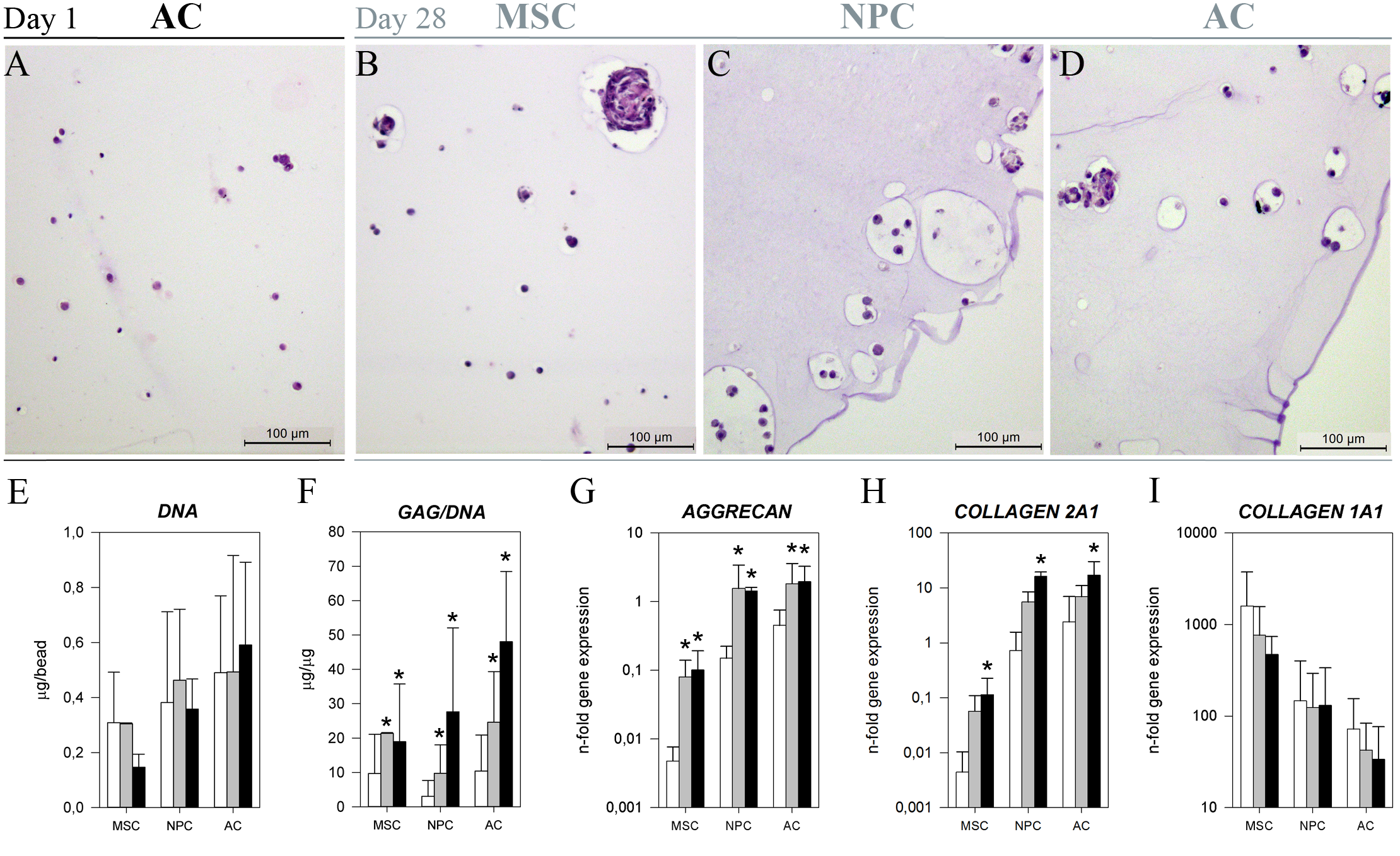

Supplement: Additional file 6: — Mesenchymal stromal cells (MSCs), nucleus pulposus cells (NPCs) and articular chondrocytes (ACs) in culture. Histopathological slides (H&E) of (A) AC on day 1 (similar to NPC and MSC on day 1). (B) through (D) Day 28 H&E staining of (B) MSCs, (C) NPCs and (D) ACs (scale bar = 100 μm). The (E) DNA content, (F) glycosaminoglycan content normalized to DNA (GAG/DNA) and gene expression of (G) aggrecan, (H) collagen type 2, α1, and (I) collagen type 1, α1. White bar = day 1, gray bar = day 15, black bar = day 28. *P < 0.01, significantly different from day 1. [file 13075_2015_569_MOESM6_ESM.tiff]
